# The Impact of Foreign Direct Investment on the Forestry Industry Structure Upgrading: The Moderating Effect on Labor Migration

**DOI:** 10.3390/ijerph20032621

**Published:** 2023-02-01

**Authors:** Fangmiao Hou, Xiaoyi Li, Chengliang Wu, Yufei Yin, Hui Xiao

**Affiliations:** School of Economics and Management, Beijing Forestry University, Beijing 100083, China

**Keywords:** forestry foreign direct investment, labor migration, forestry industry structure upgrading, moderating effect model

## Abstract

This study examines the impact of foreign direct investment in forestry, a prominent phenomenon in China, on forestry industry structure upgrading, using comprehensive economic theory and the panel data of 27 provinces from 2003 to 2019 in China. We used fixed and moderating effect models, and regional heterogeneity tests were conducted. Our results indicate that, at the national level, foreign direct investment in forestry and labor migration promotes forestry industry structural upgrading. In addition, our results indicate that labor migration as a moderating variable weakly promotes forestry industry structural upgrading via foreign direct investment in forestry, and these effects have regional heterogeneity. Finally, different control variables also have influence on forestry industry structural upgrading, such as the number of forestry stations. Based on these empirical results, we provide an explanation and give policy implications, such as developing secondary and tertiary forestry industries, building forestry infrastructure, and improving the efficiency of forestry foreign investment utilization to promote the optimization of the forestry industry structure in China.

## 1. Introduction

Forestry is a fundamental industry in China’s economic and social system and has an important impact on achieving sustainable economic growth. The development and growth of the forestry industry in China not only plays an important role in realizing rural revitalization, reducing the income gap between urban and rural areas, and promoting high-quality economic development, but it also has an important impact on achieving a balance between economic and ecological benefits, as well as helping carbon emission reduction and carbon neutrality [1]. In recent years, China’s forestry industry has developed rapidly, with the total forestry output value rising from RMB 106.52 billion in 2006 to RMB 807.51 billion in 2019, nearly tripling the output value in just over a decade. The forestry industry has the dual functions of providing forest products and ecological services. However, the current forestry industry economy is also facing various development bottlenecks and challenges; in particular, industrial structural issues are more prominent. Given that China is facing labor migration and constantly absorbing FDI, the choice of forestry industry structure upgrading is the key point of the study, in addition to the studying the factors influencing forestry industry structure upgrading. Both of these factors are in line with China’s national conditions, and are of great theoretical and practical significance. To a certain extent, this paper enriches the theoretical connotation of foreign direct investment in forestry, how this affects the upgrading of forestry industry structure, and provides a more comprehensive understanding of the influence of capital and labor on the upgrading of China’s forestry industry structure. This study also enriches the understanding of the development status of foreign direct investment in forestry and provides a more macroscopic understanding of the developmental trends and influencing factors of rural labor migration in China.

The optimization of forestry industry structure cannot be achieved without the use of foreign capital. There are four main forms of foreign investment in forestry: loans from international financial organizations, which are mainly used to support forestry development in poor areas; loans from foreign governments, which are mostly used for ecological forestry construction projects in China; non-reimbursable aid, which is mainly used to support ecological reforestation, technology development, and promotion projects; and foreign direct investment in forestry (hereafter referred to as FDI in forestry), which is mainly invested in industrial raw material forest bases and forest industry construction projects. The share of FDI in forestry has dominated among the methods of utilizing foreign investment in China’s forestry industry [2].

FDI in China’s forestry has gone through roughly three stages. The first stage being the initial cooperation stage from 1985 to 2000, which mainly involved the construction of timber forests, supplemented by the development of economic forests, bamboo forests, and multifunctional protection forests. The second stage being the steady development stage from 2001 to 2010, when international loans were mainly used for sustainable development, biodiversity, and combating climate change, and began to focus on ecological development. The third stage is the in-depth cooperation stage from 2011 to 2020, when the scale and application fields of foreign investment in forestry were expanded and the focus on forestry cooperation with foreign financial capital began. In view of the special nature of the forestry industry, FDI in forestry not only brings economic development, but also affects the ecology and institutions in a subtle way. There are many factors that affect forestry industry structure upgrading: firstly, technological progress; Jiang et al. (2021) [3] empirically tested the impact of forestry technological progress and pointed out that, within China, forestry technological progress has a significant positive contribution to the upgrading of forestry industry structure. Based on the mediating effect of industrial structure upgrading, Jiang and Wang (2022) [4] found that the biggest obstacle factor affecting the high-quality development of the forestry economy, as well as the promotion of industrial structure upgrading, is technological change. Secondly, for policy reasons, Shi et al. (2021) [5] estimated the impact of carbon sink afforestation projects (CSAP) on industrial structure upgrading via VTT-DID and integrated control methods, and the results showed that CSAP can improve the advanced level of industrial structure. Yang et al. (2014) [6] empirically tested the relative optimization of industrial structures within the primary and tertiary forestry industries as a result of the collective forest tenure reform in 2003. Other scholars used the industrial structure hierarchy coefficient method to measure the value of China’s forestry industry structure and found that the level of urbanization had a significant positive impact on the optimization of forestry industry structure [7]. Most studies have concluded that national forestry policies, ecological protection measures (such as reforestation), optimization of agricultural industry structure, economic development, and the use of domestic and foreign investment are the main factors influencing the structural upgrading of the forestry industry. However, these factors have heterogeneity in the direction of influence on provinces with different upgrading levels; thus, the optimization of the forestry industry structure should be carried out according to the characteristics of the east, middle, and west regions of China [8]. Many scholars have only focused on one of the influencing factors, or a few influencing factors alone, and less on the interactions among them. When previous studies have examined the role of FDI in forestry on the structural upgrading of China’s forestry industry, they have also been less integrated based on the context of rural labor migration. The innovation of this study is mainly reflected in the following aspects: in terms of research content, and differing from most previous studies, we have simultaneously examined the impact of labor migration and FDI on forestry industry structure upgrading. Secondly, from the perspective of research methods, we used the fixed-effect and moderating effect models to enrich the research on the influencing factors and specific influencing mechanism of the forestry industrial structure, in addition to providing a reference for the more balanced and coordinated development of China’s forestry. We have also provided policy implications for China’s forestry to utilize foreign capital more efficiently. Finally, from the perspective of research, we conducted a regional heterogeneity analysis on the basis of applying the above research framework to the forestry field, so as to more deeply clarify the influencing factors and mechanisms of the upgrading of the forestry industrial structure, and we further explored the reasons for these differences, so as to make the countermeasures and suggestions more comprehensive and targeted.

Based on the above analysis, and in the context of China’s labor migration and the continuous absorption of FDI, this article puts forward the following questions: What are the effects of FDI and labor migration on forestry industry structure upgrading, respectively? How do these two factors work together to promote forestry industry structure upgrading? What roles do other factors play in restructuring the forestry industry? Do these factors differ from one region to another in China? Based on the reality of the situation, we attempt to investigate the mechanism of FDI in forestry industry structure upgrading and how both FDI and labor migration interact to influence forestry industry structure upgrading in the context of increased rural labor outflow.

## 2. Theoretical Analysis and Research Hypothesis

The American economists John C. H. Fei and Gustav Ranis developed a new binary economic model derived from the Lewis model. This theory argued that the increase in productivity of traditional agriculture generates surplus products, which is the root cause of labor migration in the agricultural sector. Kaname Akamatsu, a Japanese scholar, proposed the flying geese model. The industrial turnover process of import–production–export can be drawn in the shape of a “V” (also known as “the flying geese”), in which the host country relies on imports in the early stage, attracts foreign capital and advanced technology, gradually develops domestic industries, then forms import substitutions, and even promotes exports to optimize the industrial structure. The change of industrial structure in the same country also follows this theory, as industries change from consumer goods to means of production, thus optimizing the industrial structure. Therefore, based on these previous theories, we propose a new theoretical framework and research hypotheses.

### 2.1. The Impact of FDI in Forestry on the Forestry Industry Structure Upgrading

The introduction of foreign capital in forestry can optimize the forestry industry structure in the region, specifically in two aspects: direct effect and indirect effect. The direct effect refers to the fact that FDI in forestry enters the production field directly or indirectly through capital input, which directly solves the problem of insufficient capital faced in the development of forestry industry and accelerates capital formation. Thus, foreign capital promotes regional economic growth and optimizes the forestry industry structure. The indirect effect refers to the fact that FDI in forestry affects regional forestry industry structure upgrading through technology migration routes, all while bringing in capital. FDI in forestry also brings in advanced technology and management experience through the correlation effect and demonstration effect, introducing advanced technology and management experience and accelerating the process of domestic technological progress, thus promoting the forestry industry from a low-tech, low-value added state to a high-tech, high-value added state, achieving optimization and upgrades within and among the three industries [9].

**Figure 1:**
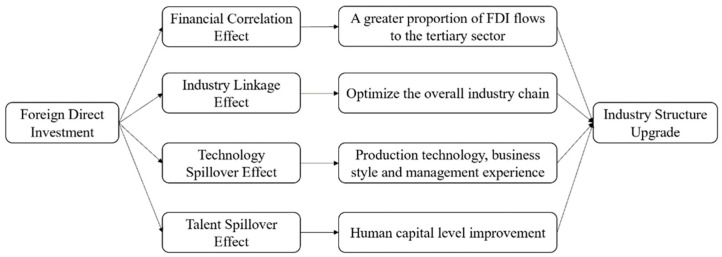
Influence mechanism of FDI in forestry on the forestry industry structure upgrading.

There are four main indirect ways for FDI in forestry to upgrade the forestry industry structure, as shown in Figure 1. The first one is capital-related effect, since the tertiary industry of forestry requires more capital investment, plus the flow of FDI in forestry itself has structural inclination to the second and third industries. Therefore, a larger proportion of FDI in forestry flows to the second and third industries. The second one is industry-related effect; when FDI in forestry enters China, it will make multinational companies and local enterprises in China more closely linked, and this linkage includes forward linkages and backward linkages [10]. In the process of such linkages, higher-quality intermediate products from developed countries can be received, better technical support can be obtained, and domestic productivity can be improved, thus optimizing the overall forestry industry chain. The third is the technology spillover effect; the inflow of foreign investment in forestry brings more advanced products and intermediate goods, while also bringing advanced production technology. The fourth is the talent spillover effect, in which FDI in forestry enhances China’s human capital level due to business contacts, staff training, and cooperative development activities [11]. In conclusion, FDI in forestry has promoted the forestry industry structure upgrading of China through the above four methods.

As a result, we propose:

**Hypothesis** **1** **(H1).** *FDI in forestry optimizes forestry industry structure upgrading.*

### 2.2. The Impact of Labor Migration on the Forestry Industry Structure Upgrading

**Figure 2:**
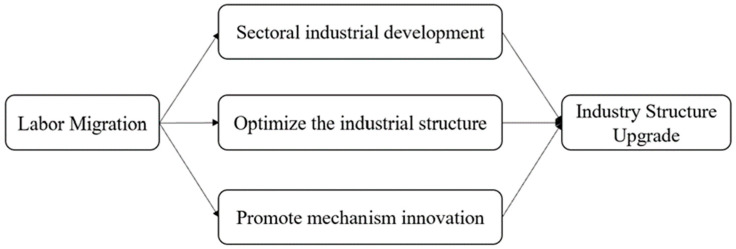
Possible paths of labor migration on the forestry industry structure upgrading.

We put forward an argument that labor migration will affect forestry industry structure upgrading in China. The influence exerted by labor migration on forestry industry structure upgrading may have the following paths (as shown in Figure 2): firstly, labor migration exerts influence on the forestry industry structure by affecting the development of industries in each sector and thus simultaneously affects the advanced forestry industry structure. When there is surplus labor in the primary industry, labor outflow will increase the productivity of the primary industry [12], and as labor flows to the secondary and tertiary industries, their productivity will also increase [13]. Secondly, labor migration optimizes the forestry industry structure by changing the labor employment structure, such as changing age structure, occupation structure, education level, technology level, etc. in order to change production efficiency, human capital level, and even consumption, thus contributing to forestry industry structure optimization. Thirdly, labor migration will also lead to the innovation of business methods and technology, which will lay the foundation for promoting forestry. Fourthly, labor migration will also drive the innovation of technology, laying the foundation for promoting forestry industry structure upgrading.

As a result, this paper proposes:

**Hypothesis** **2** **(H2).** *Labor migration plays a catalytic role in forestry industry structure upgrading.*

### 2.3. The Impact of FDI in Forestry and Labor Migration on the Forestry Industry Structure Upgrading

First, labor migration will work together with FDI in forestry industry structure upgrading. Equation (1) shows the basic form of the Cobb–Douglas production function, where *Y* is the total industrial output value, *A*(*t*) is the comprehensive technology level, *L* is the number of input labor, *K* is the input capital, α is the elasticity coefficient of labor output, β is the elasticity coefficient of capital output, and μ denotes the effect of random disturbance, μ ≤ 1. From this equation, it can be seen that the main factors that determine the development level of industrial systems are the number of input labor, fixed assets, and comprehensive technology levels (including management level, labor quality, introduction of advanced technology, etc.). Both capital and labor affect the output value, and since the secondary and tertiary forestry industries can bring more profits, an increase in output value often means that the forestry industry structure is optimized. FDI in forestry, as an important source of forestry capital, can significantly promote forestry development, while labor migration also often implies an increase or decrease in labor between industries, so both can be included in the influence system of forestry industrial structure upgrading at the same time.

(1) $$Y=A\left(t\right)L^{\alpha }K^{\beta }\mu$$

**Figure 3:**
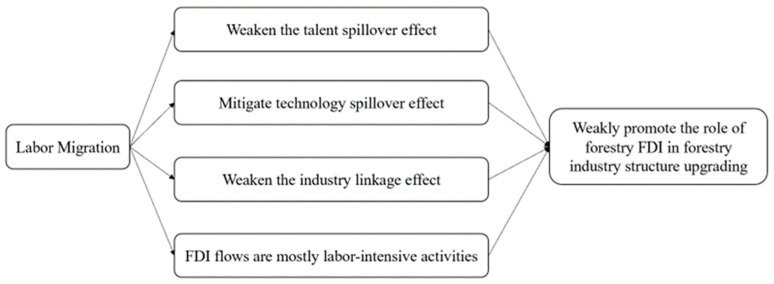
Possible impact paths of the moderating effect of labor migration.

Secondly, labor migration can lead to a weakening of the impact of FDI in forestry industry structure upgrading [14]. The specific impact pathways may be as follows (as shown in Figure 3): firstly, the most direct pathway is to weaken the talent spillover effect brought on by foreign direct investment. The highly educated labor force, as well as the young labor force, flow more to other industries when compared to forestry. The outflow of labor directly reduces forestry talents and thus slows down the improvement of human capital brought on by FDI in forestry.

Thirdly, the technology spillover effect brought on by foreign direct investment is mitigated by the outflow of labor. Due to a lack of forestry talents, the application speed of advanced technology brought by FDI is reduced. Furthermore, the industrial correlation effect between domestic forestry enterprises and transnational corporations is weakened, thus restraining the improvement of domestic productivity and the optimization of the industrial chain of forestry enterprises. Fourthly, FDI in forestry is mainly invested in labor-intensive activities such as industrial raw material forest base and forest product industrial construction projects. The migration of rural labor to cities will weaken the effect of FDI on these projects to some extent. From these aspects, labor migration weakens the promoting effect of FDI on forestry industry structure upgrading.

As a result, we propose:

**Hypothesis** **3** **(H3).** *Labor migration will weaken the promoting effect of FDI on the forestry industry structure upgrading.*

## 3. Data Indicators and Measurement Method Selection

### 3.1. Indicator Design and Data Sources

#### 3.1.1. Index Design of FDI in Forestry and Data Sources

With the globalization of the economy and the flow of production factors, the impact of foreign direct investment on various industries, including the forestry industry, has become increasingly important [10]. FDI in forestry has increased rapidly since the 1990s. This growth makes it extremely important to study the impact of FDI on the structural upgrading of the forestry industry [15]. China has benefited from globalization at a later stage by absorbing large amounts of FDI, which made it possible to introduce advanced technologies to transform and upgrade the domestic forestry industry [16]. Based on the previous definition, with reference to the study of Jiang et al. (2020) [17] on FDI in forestry, and by combining the availability of indicators, we adopted the index of FDI in forestry, and the data are directly obtained from “Actual Utilization of Foreign Investment in Forestry” in China’s Forestry Statistical Yearbook. On this basis, the data are converted from USD to RMB based on the year-end mid-rate.

#### 3.1.2. Labor Migration Indicator Design and Data Sources

The process of measuring labor migration is shown in Equations (2) and (3):

(2) $$\begin{array}{c} CL+TL=PL+IL+SL \end{array}$$

(3) $$\begin{array}{c} TRL=CL-PL=IL+SL-TL \end{array}$$

where *CL* denotes rural employed persons; *TL* denotes urban employed persons; *PL*, *IL*, and *SL* are employed persons in primary, secondary, and tertiary industries, respectively; and *TRL* is the total number of labor migration. From Equations (2) and (3), it can be seen that the difference of just division and the sum of sub-urban and rural employed persons is equal to the sum of sub-three industries employed persons, and the rural employed persons minus the primary industry employed persons is constantly equal to the secondary and tertiary industries minus the urban employed persons. This difference is used to indicate the labor force migration. In this paper, the robustness test of the regression results is conducted by using the replacement labor force indicator calculation method, and this method is chosen to calculate the labor force migration (*rTrl*) in the robustness test.

#### 3.1.3. Design of Forestry Industry Structure Upgrading Indicators and Data Sources

At present, there are five primary measures for the degree of industrial structure optimization in previous studies: the first one is the industrial structure adjustment coefficient (S1) proposed by Clark (1967) [18]. The second is the structural adjustment coefficient (S2). The third is the value of industrial structure change (*K value*). The fourth is the Moore index, and the fifth is the industrial structure hierarchy coefficient. In this paper, we chose the fifth method to measure the forestry industry structure upgrading since this calculation method is a more comprehensive measure and is reflective of the structural changes among the three industries in a timely manner. It is also more convenient to use.

Jing (2005) [19] first proposed the concept and calculation method of industrial structure hierarchy coefficient in 2005 and applied it to measure the advanced industrial structure. Many scholars believe that, compared with other methods, the industrial structure level coefficient can systematically measure the changes of primary, secondary, and tertiary industries. As shown in Equation (4), let there be *n* industries in a certain region, arrange these industries from the highest to the lowest level, and record their shares as *q*(*j*), then the industrial structure level coefficient of the region is calculated.

(4) $$\begin{array}{c} Hcofis_{it}=\overset{n}{\underset{i=1}{\sum }}\overset{i}{\underset{j=1}{\sum }}q\left(j\right) \end{array}$$

The larger the structural level coefficient of the region, the more advanced the industrial structure. The structural level coefficient reflects the comparison of the change status and degree of industrial structure advancement at different times and regions, rather than the absolute level.

Referring to Lu and Yue (2022) [20], forestry is divided into three industries, and the three industries are ranked in order of importance as tertiary, secondary, and primary industries, and assigned weights of 3, 2, and 1 to obtain Equation (5).

(5) $$\begin{array}{c} Hcofis_{it}=\overset{n}{\underset{i=1}{\sum }}a_{i}V_{i}\left(n=1,\;2,\;3\right) \end{array}$$

In Equation (5), *V_i_* represents the proportion of the added value of primary, secondary, and tertiary forestry industries to the total added value of forestry in that year, and *a_i_* represents the weight value assigned to the three industries. From the above analysis, it can be seen that the larger the value of the forestry industry structure level coefficient is, the more advanced the forestry industry is.

After designing forestry industry structure upgrading, descriptive statistics for China’s three major forestry industries for 2006–2020 are shown in Table 1 (Unit: 10,000 yuan).

During the development of China’s forestry industry from 2006–2020, the average value of the forestry primary industry is 155,874,721 with a standard deviation of 75,717,653.06 and a large degree of dispersion, with a minimum value of47,088,160 and a maximum value of RMB 263,021,121; the average value of the forestry secondary industry is 221,912,028 with a standard deviation of 115,651,907.1, a minimum value of 51,983,970, and a maximum value is RMB 364,331,594; the average value of the forestry tertiary industry is 77,531,104.73, the standard deviation is 64,574,388.82, the minimum value is 7,450,032, and the maximum value is 192,904,416. It can be seen that Chinese forestry’s three industries have increased the sub-production value, of which the primary industry of forestry accounted for a steady decline, the secondary industry accounted for an overall stable tendency, and the proportion of the tertiary industry has achieved an increase from 7,450,032 in 2006 to 1,929,040,416 in 2019, which is more than three times higher than the original. This indicates that the industrial structure of China’s forestry industry is being continuously optimized, realizing the leap from an industrial structure dominated by the primary and secondary forestry industries to an industrial structure dominated by the secondary and tertiary forestry industries. In general, in the past decade or so, the structural changes of China’s three major forestry industries are in line with Kuznets’ law of industrial upgrading.

From the previous review of the literature, we can find that the factors affecting the upgrading of the forestry industry structure mainly include national forestry policies, ecological protection measures (such as reforestation), optimization of agricultural industry structure, economic development, and the use of domestic and foreign investment.

#### 3.1.4. Selection of Control Variables and Data Sources

In this paper, the control variables are divided into two parts: one part indicates the macro factors of the regional economy, which are reflected through indicators such as GDP per capita and population size. Specifically, GDP per capita mainly measures the economic level of a region, and this may optimize the structure of the forestry industry [4]. Population size is an important driver of consumption, and the larger its value, the more likely it is to promote the development of forestry’s secondary and tertiary industries, thus optimizing the forestry industry structure [21].

The other part is the regional forestry development trait, which is reflected by the amount of forestry’s fixed investment, the number of forestry employees, the number of forestry stations, and the forest coverage rate. Specifically, the amount of FDI in forestry’s fixed assets reflects the local government’s investment in forestry, and the use of funds promotes the optimization of forestry industry structure [22]. The number of forestry employees measures the level of regional forestry labor input; the higher the labor input, the better the quality, and the more conducive it is to optimizing forestry industry structure. However, the higher number of state-owned enterprise employees plays a certain inhibiting role instead [23]. The number of forestry stations reflects the implementation of national policies and the forestry management level of local government. Hence, the more stations there are, and the higher the management level and scientific and technological investment in the region, the more likely it is that the forestry industry structure will be optimized [24]. Forest cover is an important variable to measure the endowment of forest resources in a region, and different regions have different forestry resources due to climate, topography, and precipitation, which is also the basis on which forestry is developed [25].

Xiong et al. (2018) [8] introduced GDP per capita and population size, which measure macroeconomic levels; R&D internal funding expenditure, which measure innovation and technological progress; and the number of forestry stations and forest cover, which measure the characteristics of forestry development, as control variables in the study of forestry industry structure impact system, and investigated the correlation between these indicators and forestry industry structure upgrading. According to Jiang (2021) [3], the indicators affecting the upgrading of forestry industry structure are forestry labor input, forestry capital input level (the proportion of forestry capital input to total forestry output value is used to measure the level of forestry capital input), forest resource status (the forest cover rate indicator is used to evaluate the forest resource status), and regional economic level (the GDP per capita indicator is used to evaluate the regional economic level). Based on the synthesis of previous literature and the availability of data, the selected control variables are shown in Table 2.

#### 3.1.5. Description of Variable Indicators

After the previous introduction, the selection and naming of the variables, units, and data sources are summarized in Table 3.

Since there is a significant lack of data for some provinces, only the data from 2003–2019 of 27 provinces except Hong Kong, Macao, Taiwan, Tianjin, Shanghai, Qinghai and Tibet are sorted out. The missing data of some years are configured through linear interpolation, and the amount of labor force migration in 2018–2019 is derived from 2017 on the basis of the growth rate of 2016–2017. The data are obtained from national and provincial Forestry Statistical Yearbooks, China Statistics Yearbooks, and Labor Statistical Yearbooks.

### 3.2. Descriptive Statistics of Variables

After reviewing the data and calculations, the descriptive statistics of each variable are shown in Table 4.

The dependent variable forestry industry structure upgrading coefficient (*Hcofis*) has a mean of 1.63 and a standard deviation of 0.68, with a small degree of dispersion, all located between 1.01–14.95, overall. The core independent variable FDI in forestry has a mean of RMB 220,244,100 and a great standard deviation of RMB 71,433.72, both of which are located between RMB 13.07–807,769.06. Labor migration (*Trl*) averaged 738.50 million with a standard deviation of 736.32, and also had a large degree of dispersion, lying between 34.10 and 5408.20 million overall. There are six control variables, and GDP per capita (*Pgdp*), which measures the economic level of a region, has a mean value of RMB 36,352.39 and a great standard deviation of RMB 24,430.28, and the overall lies between RMB 3603.00 and 164,222.00, with a slightly larger degree of dispersion, indicating that the level of economic development varies between regions, which requires regional heterogeneity analysis in the following regressions. The mean value of population size (*P*) is 48,161,600, with a standard deviation of 2652.37 and a large degree of dispersion, and the overall lies between 580.00–124,890,000, indicating that there are significant differences in regional size and also side-by-side, indicating the necessity of regional heterogeneity analysis. The mean value of investment in forestry fixed assets (*Iiffa*) is RMB 294,322.82 million, with a great standard deviation of RMB 1,066,611.70, and the overall lies between RMB 425.00–9,475,620.00 million. The number of forestry employees (*Fw*) has a mean value of 46,667 and a standard deviation of 58,916.37, with a large degree of dispersion, and the sample as a whole is located between 4721–378,802, which indicates that different regions have large differences in the level of investment in forestry human capital. The average number of forestry stations (*Nofs*) is 49,225 with a great standard deviation of 129,525.14. The dispersion of the data is large and the overall lies between 0–1,375,388, which indicates that there are large differences in the level of forestry management and scientific and technological investment among different regions. The average forest cover (*Fc*) is 33.05%, with a standard deviation of 16.80 and a large degree of dispersion. The overall lies between 2.94% and 66.80%, which indicates that there is a large gap in forest land resource endowment between different regions.

### 3.3. Multicollinearity Test

Collinearity refers to the non-independence of predictor variables, usually in regression-type analyses [26]. Correlated predictor variables—and potential collinearity effects—are a common concern in interpretation of regression estimates [27]. Collinearity describes the situation where two or more predictor variables in a statistical model are linearly related [28]. If multicollinearity is not dealt with, it will lead to large variance of parameter estimators and affect the validity of the model. In this paper, the variance inflation factor method is used to verify the presence of multicollinearity among the explanatory variables. The results showed that the mean value of VIF of the variables was 2.47, the minimum value was 1.10, and the maximum value was 4.73, all of which were less than 5, indicating that there was no serious problem of multicollinearity.

### 3.4. Econometric Model

The panel data model is divided into mixed regression model, fixed-effect model, and random effect model. The F-value of the F-test in the Hausman test for fixed effects was 2.97 and the *p*-value was 0.0000, so the null hypothesis was rejected in favor of the alternative hypothesis, and the fixed-effects model was selected instead of the random effects model. By testing the statistics of the regression model, the fixed-effects model was finally selected for this study. The fixed-effects model aims to solve the problem of subject heterogeneity and also eliminates the problem of bias caused by omitted variables. The Hausman test shows that it is more appropriate to use two-way fixed effects in this research, so the econometric model is mainly divided into two parts. The first part uses the fixed-effects model to conduct a benchmark regression on the influence of FDI and labor migration on forestry industry structure upgrading, with the purpose of exploring the direction of the influence of independent variables on the dependent variable, and the influence process after adding control variables, respectively. The second part uses the moderating effects model to specifically analyze how to add the variable of labor migration on the influence of the core independent variable of FDI in forestry on the dependent variable.

#### 3.4.1. Fixed-Effects Model

In order to study the impact of FDI in forestry on forestry industry structure upgrading, this paper draws on the benchmark regression method of Lin (2021) [29] to conduct a term-by-term regression, using the forestry industry structure upgrading coefficient (*Hcofis*) as the explanatory variable, FDI in forestry as the core explanatory variable, and labor migration (*Trl*) as the other explanatory variable, while including per capita GDP (*Pgdp*), population size (*P*), investment in forestry fixed assets (*Iiffa*), number of forestry employees (*Fw*), number of forestry stations (*Nofs*), and forest cover (*Fc*) as control variables. Controlling for area and time effects, the model design of Equations (6)–(8) was constructed as follows:

(6) $$\begin{array}{c} Hcofis_{it}=\alpha_{1}+\beta_{11}Fdi_{it}+\beta_{12}Province_{i}+\beta_{13}Year_{t}+\varepsilon_{it} \end{array}$$

(7) $$\begin{array}{c} Hcofis_{it}=\alpha_{2}+\beta_{21}Fdi_{it}+\beta_{22}Trl_{it}+\beta_{23}Province_{i}+\beta_{24}Year_{t}+\varepsilon_{it} \end{array}$$

(8) $$\begin{array}{c} Hcofis_{it}=\alpha_{3}+\beta_{31}Fdi_{it}+\beta_{32}Trl_{it}+\beta_{33}Province_{i}+\beta_{34}Year_{t}+\underset{j}{\sum }\varphi_{j}x_{it}^{j}+\varepsilon_{it} \end{array}$$

The subscripts *i* and *t* denote provinces/municipalities directly under the central government and years, respectively. The left side of the equal sign is the forestry industry structure upgrading coefficient $Hcofis_{it}$, which is also the explanatory variable in this study, and the forestry industry structure upgrading coefficient is used to characterize the degree of forestry industry structure upgrading. The core explanatory variables $Fdi_{it}$ and $Trl_{it}$ on the right side of the equation sign are indicators of labor migration and FDI in forestry; $\underset{j}{\sum }\varphi_{j}x_{it}^{j}$ are the control variables. *Pgdp*, *P*, *Iiffa*, *Fw*, *Nofs*, and *Fc* correspond to six control variables, namely GDP per capita, population size, investment in forestry fixed assets, number of forestry employees, number of forestry stations, and forest cover, which are added sequentially and item-by-item in the regression model. Province and Year denote the regional effect and year effect of the province to which they belong, respectively, and are controlled in the regression. The regressions are conducted in the regression model. Finally, *α* indicates the constant term, and $\varepsilon_{it}$ indicates the random error term.

#### 3.4.2. Moderating Effect Model

In order to explore the influence of FDI in forestry and labor migration on the forestry industry structure upgrading in China, and their interaction at the same time, we chose to use the moderating effect model to include the two and their interaction terms into the regression equation, respectively. Because this research mainly considers the influence of FDI in forestry with forestry industry structure upgrading, FDI is used as the independent variable and *Trl* is used as the moderating variable, while the following control variables are added. Therefore, Equations (9)–(11) are constructed to verify the effects of FDI in forestry and labor migration on the forestry industry structure upgrading.

(9) $$\begin{array}{c} Hcofis_{it}=\alpha_{4}+\beta_{41}Fdi_{it}+\beta_{42}Province_{i}+\beta_{43}Year_{t}+\varepsilon_{it} \end{array}$$

(10) $$\begin{array}{c} Hcofis_{it}=\alpha_{5}+\beta_{51}Fdi_{it}+\beta_{52}Trl_{it}+\beta_{53}Trl_{it}*Fdi_{it}+\beta_{54}Province_{i}+\beta_{55}Year_{t}+\varepsilon_{it} \end{array}$$

(11) $$\begin{array}{c} Hcofis_{it}=\alpha_{6}+\beta_{61}Fdi_{it}+\beta_{62}Trl_{it}+\beta_{63}Trl_{it}*Fdi_{it}+\ldots +\underset{j}{\sum }\theta_{j}x_{it}^{j}+\varepsilon_{it} \end{array}$$

The subscripts *i* and *t* denote provinces/municipalities directly under the central government and years, respectively. The left side of the equal sign is the forestry industry structure upgrading coefficient $Hcofis_{it}$, which is also the explanatory variable in the research. The core explanatory variables $Fdi_{it}$ and $Trl_{it}$ are indicators of labor migration and FDI in forestry, $Trl_{it}*Fdi_{it}$ is the interaction term between labor migration and FDI in forestry, and $\underset{j}{\sum }\theta_{j}x_{it}^{j}$ are the control variables. Province and Year denote the regional effect and year effect of the province they belong to, respectively, and both are controlled in the regression analysis; α denotes the constant term and $\varepsilon_{it}$ denotes the random error term.

The moderating effect means that the effect of *Trl* on *Hcofis* is moderated by the *FDI* variable, i.e., the presence of *FDI* affects the strength of the relationship between *Trl* and *Hcofis*. In the model, Equation (9) is the main effect, Equation (10) adds the moderating variable *Trl* term to Equation (11) and adds the interaction term Trl*Fdi between the moderating variable *Trl* term and the main effect *Fdi* term. If $\beta_{41}$ is significant, i.e., the main effect is significant, while $\beta_{53}$ is significant, i.e., the interaction term is significant, it indicates that there is a moderating effect. In case the results hold, if $\beta_{41}$ and $\beta_{53}$ have the same sign, the moderating variable *FDI* strengthens the main effect; if $\beta_{41}$ and $\beta_{53}$ have opposite signs, the moderating variable *FDI* weakens the main effect. Specifically, at $\beta_{41}$ > 0, if $\beta_{53}$ > 0, the moderating variable *FDI* strengthens the promoting effect of *Trl* on *Hcofis*, and if $\beta_{53}$ < 0, the moderating variable *FDI* weakens the promoting effect of *Trl* on *Hcofis*; at $\beta_{41}$ < 0, if $\beta_{53}$ > 0, the moderating variable *FDI* weakens the promoting effect of *Trl* on *Hcofis*, and at $\beta_{41}$ < 0, if $\beta_{53}$ < 0 the moderating variable *FDI* strengthens the inhibitory effect of *Trl* on *Hcofis*.

## 4. Estimated Results

### 4.1. Estimated Results of FDI in Forestry and Labor Migration on Forestry Industry Structure Upgrading

Model 1 in Table 5 illustrates the regression results for Equation (6), and it can be seen that, without adding any other variables, the regression results of FDI in forestry on the hierarchical coefficient of forestry industry structure (*Hcofis*) are significantly positive and significant at the 5% statistics level. Models 2–8 show that the other independent variable, labor migration (*Trl*), and each control variable were gradually added to the regression for the baseline models of Equations (7) and (8), and the results are shown in Table 6.

As shown in Models 2–8 of Table 6, with the influence coefficient of the core explanatory variable FDI on forestry industry structure upgrading, the coefficient (*Hcofis*) is still significantly positive after gradually adding control variables, which indicates that the influence of FDI on forestry industry structure upgrading is positive and stable in trend, and hypothesis 1 holds. The impact coefficient of the FDI in forestry industry structure upgrading coefficient (*Hcofis*) in Model 8 is 0.0096 and is significant at the 10% level, indicating that for every RMB 10,000 increase in FDI in forestry, the forestry industry structure upgrading coefficient (*Hcofis*) will expand by about 0.010, which approximates to 0.01. The reasons for this may lie in the following three aspects: first, foreign enterprises established through FDI can provide local enterprises with higher quality intermediate inputs or stimulate the development of local enterprises through procurement and other means, thus optimizing the industrial structure [30]. Second, the products and technologies brought by foreign enterprises can reduce the R&D costs of enterprises and improve the operational efficiency of local enterprises, thus promoting industrial restructuring. Third, the level of human capital is improved through the movement of personnel and invariably improves the working ability of relevant R&D personnel to drive the optimization and upgrading of the industrial structure. The empirical results have proved hypothesis 1. FDI in forestry can solve the problem of capital shortage faced by forestry industry development, promote regional economic growth, and optimize forestry industry structure through direct and indirect effects. Based on four ways, capital-related effect, industry- related effect, technology spillover effect and talent spillover effect, the upgrading of China’s forestry industry structure is promoted.

As shown in Model 2, the regression result of the other independent variable, labor migration (*Trl*), on the forestry industry structure upgrading, which was first included in the model, is positive and significant at the 10% level, and the coefficient is still significantly positive after gradually adding control variables to the regression. The effect of labor migration (*Trl*) on the forestry industry structure upgrading (*Hcofis*) in Model 8 is 1.142 and is significant at the 5% level, indicating that the forestry industry structure upgrading (*Hcofis*) will expand by about 1.142 for every 10,000 people increase in labor migration. The reason may be that the outflow of well-educated labor promotes the innovation of the forestry industry and the development of tertiary industries such as forest recreation and eco-tourism, thus promoting the advancement of the forestry industry. Additionally, it may be that the flow of labor improves economic efficiency, promotes “job matching”, and promotes the improvement of labor productivity, thus promoting forestry industry structure upgrading. This conclusion is consistent with most previous studies [31]. The empirical results have proved hypothesis 2. Labor migration can lay the foundation for promoting forestry development by influencing the development of industries in various sectors of forestry, which in turn affects the advanced structure of forestry industry, changing the employment structure of the labor force and bringing about innovations in business practices and technologies.

As for the control variables, after gradually adding control variables to the baseline model, the final results are shown in Model 8, and only the regression result of the number of forestry stations (*Nofs*) on the forestry industry structure upgrading (*Hcofis*) is significantly positive and significant at the 10% level with a regression coefficient of 0.0008. This indicates that for every 1% increase in the number of forestry stations, the forestry industry structure upgrading coefficient will expand by about 0.0008. This generally indicates that forestry stations can characterize the degree of state management of forestry in the forest area and the implementation of forestry policies in the area, both in terms of technical support and state management to promote the forestry industry structure upgrading. The regression results of the control variables GDP per capita (*Pgdp*), population size (*P*), investment in forestry fixed assets (*Iiffa*), number of forestry employees (*Fw*), and forest cover (*Fc*) on the hierarchical coefficient of forestry industry structure (*Hcofis*) were not significant.

### 4.2. Estimated Results of the Moderating Effect of FDI in Forestry and Labor Migration on the Forestry Industry Structure Upgrading

Models 9–16 analyze the moderating effect of FDI in forestry on the relationship between labor migration and forestry industry structure upgrading. The regression results of Model 9 in Table 7 illustrate that, without adding any other variables, the main effect is significantly positive, which indicates that FDI in forestry is a prominent influence on the optimization of the forestry industry structure. Model 10 shows that the other independent variable, labor migration (*Trl*), the interaction term between labor migration and FDI in forestry ($Trl_{it}*Fdi_{it}$), and the control variables are gradually added to the regression for the baseline model of Equations (10) and (11), and the regression results are shown in Table 7.

Model 10 of Table 7 shows that the coefficient is significantly negative after adding the interaction term, and after adding the control variables one-by-one, the coefficient of Model 16 is still negative and stable, with the opposite sign of the main coefficient, which indicates that labor migration as a moderating variable weakens the role of FDI in forestry in promoting the forestry industry structure upgrading. This may be due to the fact that one of the major flows of FDI in forestry is industrial raw material forest base and forest industry construction projects, which are labor-intensive activities, and the rural labor migration to urban areas will to some extent weaken the effect of FDI on these projects, thus weakening the effect of FDI in forestry on the optimization of forestry industry structure.

As for the control variables, after gradually adding control variables to the baseline model, the final results are shown in Model 16, and only the regression of the number of forestry stations (*Nofs*) on the forestry industry structure upgrading (*Hcofis*) has a significant positive influence and is significant at the 10% level with a regression coefficient of 0.001. This indicates that for every 1% increase in the number of forestry stations, the coefficient of forestry industry structure upgrading will expand by about 0.001. This is consistent with the conclusions of Xiong (2018) [8], indicating that overall forestry stations can characterize the degree of state management of forestry in that forest area and the implementation of forestry policies in that area, both in terms of technical support and state management to guide the forestry industry structure upgrading. The regression results of Model 16 indicate that the control variables GDP per capita (*Pgdp*), population size (*P*), investment in forestry fixed assets (*Iiffa*), number of forestry employees (*Fw*), and forest cover (*Fc*) have no significant influence on forestry industry structure (*Hcofis*), which is not consistent with the conclusions of Xiong (2018) [8]. This may be due to the difference in results, which may be caused by the different time spans adopted for the research data. However, this conclusion is consistent with the baseline regression. The empirical results have proved hypothesis 3. Labor migration can weaken the impact of FDI on the forestry industry structure upgrading. It will directly weaken the talent spillover effect brought by FDI, alleviate the technology spillover effect brought by FDI, and weaken the industrial linkage effect between domestic forestry enterprises and multinational companies. The migration of rural labor to cities will weaken the impact of FDI on some labor-intensive projects to a certain extent, thus inhibiting the improvement of domestic productivity and the optimization of the industrial chain of forestry enterprises. In general, at the national level, FDI in forestry promotes forestry industry structure upgrading, and labor migration weakens this effect. However, further heterogeneity analysis and robustness tests are needed to determine whether this result is applicable to different regions and whether it is affected by the calculation methods of variable indicators.

### 4.3. Heterogeneity Test Results of FDI in Forestry and Labor Migration on Forestry Industry Structure Upgrading

The regional distribution of FDI and mobile population in China is uneven, and the introduction of foreign investment is characterized by “strong in the east and weak in the west” [32], with a relatively large number of highly educated mobile populations in the eastern region, and the professional and technical personnel among the mobile population showing an “inflow from the west to the east” [33]. In this research, we intend to adopt the method of dividing China into three regions, the east, the central, and the west, for regional heterogeneity analysis, combining data availability and dividing twenty-seven provinces into three parts, among which the eastern region includes nine provinces (cities), including Beijing, Hebei, Liaoning, Jiangsu, Zhejiang, Fujian, Shandong, Guangdong and Hainan, the central region has eight provincial administrative regions, namely Shanxi, Jilin, Heilongjiang, Anhui, Jiangxi Henan, Hubei, and Hunan, and the western region includes a total of ten provincial administrative regions, namely Sichuan, Chongqing, Guizhou, Yunnan, Shaanxi, Gansu, Ningxia, Xinjiang, Guangxi, and Inner Mongolia. Heterogeneity analysis was performed on the moderated model, and the regression results are shown in Table 8.

For the eastern region, after controlling for the factors of each control variable, the regression result of FDI in forestry (*FDI*) on forestry industry structure upgrading (*Hcofis*) is positive and significant at the 5% level; that is, it shows a significant promoting effect, and the regression coefficient is 0.0267, which indicates that for every RMB 10,000 increase in FDI in forestry, the coefficient of forestry industry structure upgrading will expand by about 0.0267. After adding labor migration (*Trl*) as a moderating variable, the regression result of the interaction term Fdi*Trl is negative and significant at the 1% level, and the moderating effect holds. Meanwhile, the sign of the interaction term is opposite to the main effect, so *Trl* as a moderating variable weakens the positive effect of FDI on forestry industry structure upgrading. In other words, with the increase in labor migration, the promotion effect of FDI on forestry industry structure upgrading becomes weaker.

As for the control variables, the regression result of population size (*P*) on forestry industry structure upgrading (*Hcofis*) is positive and significant at the 5% level, with a regression coefficient of 0.142, which indicates that for every 10,000 people increase in population size, the forestry industry structure upgrading coefficient will expand by about 0.142. The regression result of investment in forestry fixed assets (*Iiffa*) on the hierarchical coefficient of forestry industry structure (*Hcofis*) is negative and significant at the 1% level, which means there is a significant inhibitory effect, and the regression coefficient is −0.241, which indicates that for every RMB 10,000 increase in forestry fixed asset investment, the forestry industrial structure hierarchy coefficient will decrease by about 0.241. The coefficient of FDI*Trl in Model 18 is −4.961, which is statistically significant at the 5% level. It is different from the coefficient of Model 17 and Model 19, which reflects the heterogeneity between regions. That is, in the central region reflected in Model 18, with the increase in labor migration, the promoting effect of FDI in forestry on the forestry industrial structure upgrading becomes the weakest.

For the central region, after controlling for the factors of each control variable, the regression result of FDI in forestry (*FDI*) on forestry industry structure upgrading (*Hcofis*) is positive and significant at the 1% level; that is, it shows a significant promoting effect. Additionally, the regression coefficient is 0.205, which indicates that for every RMB 10,000 increase in FDI in forestry, the coefficient of forestry industry structure upgrading will expand by about 0.205. After adding labor migration (*Trl*) as a moderating variable, the regression result of the interaction term Fdi*Trl is negative and significant at the 5% level, and the moderating effect holds, while the interaction term is opposite in sign to the main effect coefficient. Therefore, *Trl* as a moderating variable weakens the positive effect of FDI in forestry on forestry industry structure upgrading. In other words, with the increase in labor migration, the promotion effect of FDI on the forestry industry structure upgrading becomes weaker. This is the same as the eastern region.

As for the control variables, the regression of population size (*P*) on the forestry industry structural hierarchy coefficient (*Hcofis*) is negative and significant at the 10% level, i.e., significantly depressed, with a regression coefficient of −0.329, which indicates that for every 10,000 people increase in population size, the forestry industry structural hierarchy coefficient will decrease by about 0.329. The regression of forest cover (*Fc*) on the forestry industry structural level coefficient (*Hcofis*) has a negative sign and is significant at the 10% level, i.e., it is significantly suppressed, and the regression coefficient is −0.014, which indicates that for every 1% increase in forest cover, the forestry industry structural level coefficient will decrease by about 0.014. The regression results of GDP per capita, investment in forestry fixed assets, forestry employees, and number of forestry stations are not significant. For the western region, after controlling for the factors of each control variable, both FDI in forestry (*FDI*) and the interaction term coefficient Fdi*Trl are found to be insignificant, which indicates that the moderating effect does not hold in the western region.

As for the control variables, the regression of investment in forestry fixed assets (*Iiffa*) on the forestry industry structure upgrading coefficient (*Hcofis*) has a negative influence and was significant at the 1% level; that is, it has a significant inhibitory effect, with a regression coefficient of −0.158, which indicates that for every RMB 10,000 increase in investment in forestry fixed assets, the forestry industry structure upgrading coefficient will decrease by 0.158. The regression results for GDP per capita, population size, forestry employees, and the number of forestry stations were not significant.

The results of the heterogeneity analysis show that in the eastern and central regions, the promotion effect of FDI on forestry industry structure upgrading becomes weaker with the increase in labor migration, but this moderating effect does not hold in the western region. The reasons for this difference come from both FDI in forestry and labor migration. First, the uneven regional distribution of FDI in forestry [34] causes the technology spillover of FDI in forestry to have a greater impact on the eastern and central regions [35]. Second, less labor migration into the western region combined with less FDI causes less opportunities for labor forces to be employed in foreign-owned enterprises [36].

Heterogeneity analysis also shows that some of the control variables became significant for forestry industry structure upgrading. Specifically, the population size in the eastern region contributes significantly to forestry industry structure upgrading. This is probably because the tertiary forestry industry is more developed in the eastern region, and a larger population size can promote the development of the tertiary forestry industry, thus optimizing the forestry industry structure. On the other hand, the investment in forestry fixed assets has a significant inhibitory effect on the upgrading of the forestry industry structure, which may be due to the low return on investment in forestry. Population size and forest cover in the central region have a significant inhibitory effect on forestry industry structure upgrading. This may be due to the fact that most people in the central region are more dependent on the products of the primary and secondary forestry industries than the tertiary forestry industry.

Forest cover also shows a significant inhibitory effect on forestry industry structure, i.e., the richer the forest resources in the central region, the slower the development of higher-level forestry industries. The main reason may be that the existing forestry tertiary industry development areas, such as protected areas, forest parks, and tourist attractions, account for a smaller proportion of the national forest area. Meanwhile, the areas with the richest forest resources (the higher the forest cover) often have transportation and infrastructure construction that cannot keep up with the demand for high-level development. The relationship between the other control variables and the forestry industry structure upgrading is not significant. The effect of forestry fixed asset investment on forestry industry structure upgrading in western regions is significantly inhibited, which may be due to the low return of current forestry investments. The effect of forest cover on forestry industry structure upgrading is significantly promoted, probably due to the fact that the high-level forestry industry in the western region is not developed. Additionally, the high forest cover provides the possibility of developing a high-level forestry industry. The relationship between other control variables and forestry industry structure upgrading was not significant.

### 4.4. Robustness Test Analysis of FDI in Forestry and Labor Migration on Forestry Industry Structure Upgrading

To make the regression results more robust, we refer to the research of He et al. (2011) [37] and use the difference between the number of people employed in secondary and tertiary industries and those employed in urban areas to indicate the number of labor migration (*rTrl*) to perform a robustness test. The robustness test of the baseline regression is conducted again using this indicator by substituting into Equations (6)–(8), and Table 9 shows the new regression results obtained.

After controlling for the factors of each control variable, the regression results of FDI in forestry (*FDI*) on the forestry industry structure upgrading (*Hcofis*) were positive and significant. The regression results of labor migration (*Trl*) were also significantly positive, which is consistent with the findings of the benchmark regression. The regression coefficients of the six control variables on forestry industry structure upgrading were not significant; that is, the correlation effect was not significant, while the regression results of the number of forestry stations in the benchmark regression were significantly positive, which was slightly different from the benchmark regression. In conclusion, the baseline regression passed the robustness test and did not affect the main conclusions of the study. The robustness test of the moderating effect was performed again, using this indicator by substituting into Models (9)–(11), and Table 10 shows the new regression results obtained.

After controlling for the factors of each control variable, the regression results of FDI in forestry (*FDI*) on forestry industry structure upgrading (*Hcofis*) are all significantly positive, and the regression results of the interaction term Fdi*rTrl are significantly negative, which is consistent with the previous conclusions. The regression coefficients of the six control variables on forestry industry structure upgrading are not significant. The relevant effects are not significant, while the regression results of some of the control variables in the previous manuscript are significant, which is slightly different. In conclusion, the moderating effect passed the robustness test, and the effect of labor migration on the effect of FDI in forestry industry structure upgrading is robust.

## 5. Discussion

This study aimed to address whether foreign direct investment affects forestry industry structure upgrading. Based on the influence mechanism of FDI in forestry on the forestry industry structure upgrading, we conducted the study in China, enabling us to take advantage of the fixed-effects model and moderating effect model in exploring the relationship between foreign direct investment, labor migration, and forestry industry structure upgrading.

The contributions of this study are mainly in the following three dimensions: first, unlike most previous studies [15,38], we examined both the effect of foreign direct investment and the moderating effect of labor migration on forestry industry structure upgrading. The moderating effect is related to the estimation of the interaction term in moderating effect Models (10)–(11). The overall effect is measured by the coefficient of forestry industry structure upgrading.

It is generally found that foreign direct investment has a significantly positive effect on forestry industry structure upgrading. This is consistent with previous studies, and the results confirm both our hypothesis and previous studies; that FDI promotes the forestry industry structure upgrading [39,40]. However, this study’s results differ from the others [4,27] which do not consider the moderating effect of labor migration. This study reports that foreign direct investment and labor migration promote forestry industry structure upgrading, and this promotion effect passes the robustness test. We mainly analyzed the influences on forestry industry structure upgrading from the dimension of the moderating effect of labor migration. Our results also indicate that labor migration as a moderating variable weakens the promotion effect of foreign direct investment in forestry industry structure upgrading. By conducting a heterogeneity analysis of the moderating model, we also verified that the effect of labor migration and FDI in forestry industry structure upgrading is robust. However, it is difficult for us to capture the moderating effect of labor migration in each area.

Second, previous studies have not considered the impact of changes in labor migration under certain socioeconomic conditions on forestry industry structure upgrading [8]. Thus, the moderating effect of labor migration has been added. At present, labor migration is one of the most important livelihood strategies for rural households in China. Therefore, it is crucial to consider labor migration as a prominent factor to analyze the impact of forestry industry structure upgrading.

Third, these results provide a new perspective on how factors such as labor migration and FDI in forestry affect forestry industry structure upgrading, complementing previous findings that FDI in forestry affects forestry industry structure upgrading [15,38]. The research in this paper contributes to a deeper understanding of the moderating effects of FDI and labor migration on the structure of the forestry industry. This research enriches and complements the analytical framework based on a new dual economic background, combined with data from a nationwide sample.

There are several ways to explain the conclusive discrepancies: first, former studies have not considered the effect of labor migration. Most previous studies used the regression effect of FDI in forestry, but this conclusion has not considered the change in human capital. Second, previous studies have not used the data from different terrains and areas, yet terrain conditions, social conditions, and income levels in different regions will affect forestry industry structure upgrading. This is partly because time effect and area effect are important variables.

These results provide new insights into how the moderating effect of labor migration affects forestry industry structure upgrading, and therefore complements the former conclusions that FDI in forestry affects the forestry industry structure upgrading. This study contributes to the literature by improving our understanding of the influence of FDI in forestry and labor migration on the forestry industry structure upgrading. Additionally, the research combines fixed-effects model and moderating effect model methods to reveal factors associated with forestry industry structure upgrading.

Even though this study has contributed to an improved understanding of the relationship between FDI in forestry and labor migration into the framework of influencing factors of forestry industry structure upgrading, there still exist some shortcomings that need further research.

First, this study only focuses on sample areas in China. Our results may be different from those obtained in other parts of the world. Other regions in other countries have different forestry industry development statuses. Therefore, we suggest that other studies should extrapolate our conclusions to other regions where forest resources, socioeconomic conditions, demographic, and institutional characteristics might be different, since the question of whether the conclusions from this study can be applied to other regions needs to be further proved. Thus, our findings need to be interpreted with caution. More efforts should be made to examine variations in forestry industry structure upgrading between regions with different provincial characteristics, as well as socioeconomic characteristics.

Second, FDI in forestry is a broad concept, which has many facets, such as sino-foreign joint ventures, sino-foreign cooperative enterprises, wholly foreign-owned enterprises, and cooperative development. In order to fully capture the impacts of FDI in forestry and labor migration on forestry industry structure upgrading, it is worthwhile for future research to further explore the impact of these and other elements of FDI in forestry and labor migration on forestry industry structure upgrading. We hope that future research can be carried out from this perspective.

In terms of future research outlooks, firstly, in measuring forestry industry structure upgrading, the indicator of a forestry industry structure level coefficient is used, which only reflects the heightened, but not the rationalized, forestry industry structure. Therefore, we can start from this aspect to do further research on the influencing factors and theoretical mechanisms of forestry industry structure upgrading in a more comprehensive way.

Secondly, the technological innovation factor can also be included in the selection of control variables, since foreign direct investment in China is mainly utilized through the promotion of innovation or technology spillover effects to promote industrial development. Therefore, the innovation factor or technology spillover factor should be included in the factors influencing the upgrading of forestry industry structure to make a more comprehensive consideration.

Third, in terms of research methods, the choice of empirical models is slightly simpler. Other possible influence paths of FDI in forestry and labor migration in influencing forestry industry structure upgrading can be explored, and the problem of endogeneity that exists in the model has not been properly dealt with. In addition, in terms of robustness testing, different robustness testing methods could be used to make the conclusions more reliable.

## 6. Conclusions

With the increasing outflow of labor migration from rural areas, the interaction between FDI in forestry and labor migration has had a positive impact on forestry industry structure upgrading. This study aimed to address whether FDI in forestry and labor migration affected forestry industry structure upgrading. Based on the dual economy model, we used a panel data of 27 provinces from 2003 to 2019 to explore the effects of FDI in forestry and labor migration on forestry industry structure upgrading. We also conducted a fixed-effects regression, moderating effects regression, and robustness test on the collected data. Our results indicated that: (1) FDI in forestry and labor migration promote forestry industry structure upgrading, and this promotion effect passes the robustness test. (2) Labor migration as a moderating variable weakens the promotion effect of FDI in forestry industry structure upgrading. (3) The heterogeneity analysis shows that this moderating relationship is regionally heterogeneous and holds only in the eastern and central regions, but not in the western region. (4) In general, the number of forestry stations has a certain role in promoting the optimization of forestry industry structure, but the relationship between the variables characterizing economic development, GDP per capita, population size, and the variables characterizing forestry development traits, forestry fixed asset investment, forestry employees, forest cover, and the forestry industry structure upgrading is not significant, and there is regional heterogeneity in the role of control variables on the forestry industry structure upgrading.

## 7. Implications

The following implications are made in response to the above results. Firstly, the Chinese government should balance the regional distribution of FDI and accelerate forestry industry structure upgrading. It should formulate and implement differentiated regional-oriented policies to coordinate the development of forestry industries in the eastern, central, and western regions, including increasing foreign investment in secondary forestry industries in the central and western regions and advancing infrastructure construction in the western regions. The Chinese government should also introduce foreign investment in the eastern regions to develop tertiary forestry industries.

Secondly, while introducing foreign investment, attention should also be paid to the level of human capital, the degree of trade openness, the level of scientific and technological development, and the scientific formulation of government policies in the country. The relevant forestry departments can further improve the investment in forestry science and technology, such as strengthening the construction of forestry stations, increasing R&D funding support for industries such as wood processing in the secondary sector and forest tourism in the tertiary sector, and using a good development environment to attract FDI and maximize its technological spillover effect. This will aid in the realization of technological progress to promote the optimization of the forestry industry structure.

Thirdly, the government should take certain policy measures to ensure that labor migration and economic growth are in harmony. It should lower the cost of labor migration, gradually remove the barriers to labor migration between regions and industries, and provide more technical training to the relevant personnel to increase the possibility of surplus labor migration and induce efficient labor migration.

## Figures and Tables

**Table 1:** Descriptive statistics table of China’s three major forestry industries.

Variable Name	Average	Standard Deviation	Minimum Value	Maximum Value
*Industry_p_*	155,874,721	75,717,653.06	47,088,160	263,021,121
*Industry_s_*	221,912,028	115,651,907.1	51,983,970	364,331,594
*Industry_t_*	77,531,104.73	64,574,388.82	7,450,032	192,904,416

**Table 2:** Table of control variables.

Control Variables	Unit	Representational Meaning
GDP per capita	Yuan	Level of regional economic development
Population scale	Million	Regional scale
Forestry fixed asset investment	Million	Level of capital input in forestry
Forestry employees	Person	Forestry labor input level
Number of forestry stations	Individual	Forestry management status
Forest coverage rate	%	Status of forest resources

**Table 3:** Illustrative table of variable indicators.

Classification of Variables	Meaning of Variables	Variable Symbols	Unit	Data Source
Dependent variable	Forestry industry structure upgrading	*Hcofis*	/	China Forestry Statistical Yearbook and Provincial Forestry Statistical Yearbooks
Independent variables	Labor migration	*Trl*	Per 10,000 People	Statistical Compilation of 60 Years of New China, Provincial Statistical Yearbooks, Provincial Labour Force Statistical Yearbooks
	FDI in forestry	*FDI*	Per 10,000 Yuan	China Forestry Statistical Yearbook and Provincial Forestry Statistical Yearbooks
Control variables	GDP per capita	*Pgdp*	Yuan	China Statistical Yearbook and Provincial Statistical Yearbooks
	Population scale	*P*	Per 10,000 People	China Statistical Yearbook and Provincial Statistical Yearbooks
	Forestry fixed asset investment	*Iiffa*	Per 10,000 Yuan	China Forestry Statistical Yearbook and Provincial Forestry Statistical Yearbooks
	Forestry employees	*Fw*	Person	Chinese Forestry Statistical Yearbook and Provincial Forestry Statistical Yearbooks
	Number of forestry stations	*Nofs*	/	Chinese Forestry Statistical Yearbook and Provincial Forestry Statistical Yearbooks
	Forest coverage rate	*Fc*	%	Chinese Forestry Statistical Yearbook and Provincial Forestry Statistical Yearbooks

**Table 4:** Descriptive statistics table.

Variable Name	Average	Standard Deviation	Minimum Value	Maximum Value
*Hcofis*	1.63	0.68	1.01	14.95
*FDI*	22,024.41	71,433.72	13.07	807,769.06
*Trl*	738.50	736.32	34.10	5408.20
*Pgdp*	36,352.39	24,430.28	3603.00	164,222.00
*P*	4816.16	2652.37	580.00	12,489.00
*Iiffa*	294,322.82	1,066,611.70	425.00	9,475,620.00
*Fw*	46,667.06	58,916.37	4721.00	378,802.00
*Nofs*	49,225.81	129,525.14	0	1,375,388.00
*Fc*	33.05	16.80	2.94	66.80

**Table 5:** Sample multicollinearity test.

Variable Name	VIF	1/VIF
*FDI*	1.21	0.824881
*Trl*	4.65	0.215247
*Pgdp*	4.73	0.211413
*P*	2.37	0.422696
*Iiffa*	1.10	0.904992
*Fw*	1.12	0.896800
*Nofs*	1.20	0.835519
*Fc*	1.29	0.773519
year		
2004	2.31	0.432257
2005	2.34	0.427967
2006	2.40	0.416336
2007	2.81	0.355961
2008	2.82	0.354640
2009	2.90	0.345059
2010	2.73	0.366710
2011	2.80	0.356700
2012	2.52	0.396940
2013	2.85	0.350270
2014	2.64	0.378915
2015	2.92	0.342522
2016	2.76	0.362818
2017	2.24	0.446873
2018	2.15	0.464987
Mean VIF	2.47	

**Table 6:** Estimated results of the impact of FDI in forestry and labor migration on the forestry industry structure upgrading.

	Model 1	Model 2	Model 3	Model 4	Model 5	Model 6	Model 7	Model 8
*FDI*	0.011 **	0.013 **	0.012 **	0.012 **	0.012 **	0.011 *	0.011 *	0.010 *
	(2.087)	(2.438)	(2.346)	(2.345)	(2.171)	(1.790)	(1.819)	(2.024)
*Trl*		0.809 *	0.916 **	1.013 ***	1.002 **	1.077 **	1.146 **	1.142 **
		(1.8581)	(2.339)	(2.830)	(2.345)	(2.438)	(2.488)	(2.4059)
*Pgdp*			−0.007	−0.004	−0.005	−0.011	−0.012	−0.015
			(−0.369)	(−0.227)	(−0.283)	(−0.639)	(−0.691)	(−0.814)
*P*				−0.032	−0.017	−0.029	−0.035	−0.031
				(−0.277)	(−0.153)	(−0.235)	(−0.283)	(−0.241)
*Iiffa*					−0.004	−0.003	−0.003	−0.004
					(−0.860)	(−0.711)	(−0.759)	(−0.855)
*Fw*						−0.001	−0.002	−0.004
						(−0.088)	(−0.178)	(−0.273)
*Nofs*							0.001 *	0.001 *
							(1.723)	(1.803)
*Fc*								0.003
								(0.464)
Constant	1.336 ***	1.307 ***	1.309 ***	1.454 **	1.391 **	1.454 **	1.484 **	1.391 **
	(32.432)	(31.695)	(33.214)	(2.655)	(2.551)	(2.529)	(2.550)	(2.127)
Regional effect	Yes	Yes	Yes	Yes	Yes	Yes	Yes	Yes
Time effect	Yes	Yes	Yes	Yes	Yes	Yes	Yes	Yes
N	279	279	279	279	268	256	256	256
R^2^	0.705	0.713	0.714	0.715	0.729	0.724	0.726	0.728

Note: Robust t-values in parentheses, *** *p* < 0.01, ** *p* < 0.05, * *p* < 0.1.

**Table 7:** Estimated results of the moderating effect of FDI in forestry and labor migration on forestry industry structure upgrading.

	Model 9	Model 10	Model 11	Model 12	Model 13	Model 14	Model 15	Model 16
*FDI*	0.011 **	0.023 ***	0.022 ***	0.022 ***	0.024 ***	0.022 ***	0.022 ***	0.021 ***
	−2.087	−4.036	−4.329	−4.319	−4.146	−3.414	−3.413	−2.835
*Trl*		0.846 *	0.936 **	1.031 ***	1.036 **	1.121 **	1.191 **	1.188 **
		(1.943)	(2.362)	(2.866)	(2.439)	(2.543)	(2.592)	(2.507)
FDI*Trl		−0.218 **	−0.204 **	−0.202 **	−0.252 **	−0.223 **	−0.228 **	−0.230 **
		(−2.211)	(−2.208)	(−2.254)	(−2.554)	(−2.101)	(−2.162)	(−2.388)
*Pgdp*			−0.006	−0.003	−0.004	−0.010	−0.011	−0.014
			(−0.316)	(−0.179)	(−0.229)	(−0.581)	(−0.633)	(−0.755)
*P*				−0.031	−0.016	−0.029	−0.036	−0.031
				(−0.273)	(−0.147)	(−0.237)	(−0.286)	(−0.2435)
*Iiffa*					−0.004	−0.003	−0.004	−0.004
					(−0.898)	(−0.744)	(−0.792)	(−0.889)
*Fw*						−0.001	−0.003	−0.004
						(−0.105)	(−0.195)	(−0.291)
*Nofs*							0.001 *	0.001 *
							(1.730)	(1.805)
*Fc*								0.003
								(0.460)
*Constant*	1.336 ***	1.306 ***	1.308 ***	1.451 **	1.386 **	1.453 **	1.484 **	1.389 **
	(32.432)	(31.661)	(33.138)	(2.657)	(2.558)	(2.540)	(2.563)	(2.134)
*Regional effect*	Yes	Yes	Yes	Yes	Yes	Yes	Yes	Yes
*Time effect*	Yes	Yes	Yes	Yes	Yes	Yes	Yes	Yes
*N*	279	279	279	279	268	256	256	256
*R* ^2^	0.705	0.714	0.715	0.716	0.731	0.725	0.728	0.730

Note: Robust t-statistics in parentheses, *** *p* < 0.01, ** *p* < 0.05, * *p* < 0.1.

**Table 8:** Estimated results of the regional heterogeneity test.

	Model 17	Model 18	Model 19
*FDI*	0.027 **	0.205 ***	−0.002
	(3.172)	(4.252)	(−0.040)
*Trl*	−0.104	1.300	−0.419
	(−0.168)	(1.500)	(−0.835)
FDI*Trl	−0.330 ***	−4.961 **	1.461
	(−3.745)	(−3.460)	(1.242)
*Pgdp*	0.006	−0.066	−0.002
	(0.417)	(−0.802)	(−0.060)
*P*	0.142 **	−0.329 *	−0.112
	(3.178)	(−2.022)	(−0.816)
*Iiffa*	−0.241 ***	0.009	−0.158 ***
	(−3.798)	(1.522)	(−6.572)
*Fw*	−0.023	0.004	−0.014
	(−0.761)	(0.416)	(−0.587)
*Nofs*	1.619	−0.828	−0.000
	(1.087)	(−0.503)	(−0.081)
*Fc*	0.004	−0.014 *	0.011 **
	(0.420)	(−2.155)	(2.449)
Constant	0.532	3.705 ***	1.571 **
	(1.481)	(4.087)	(2.996)
Regional effect	Yes	Yes	Yes
Time effect	Yes	Yes	Yes
N	74	83	99
R^2^	0.825	0.937	0.756

Note: Robust t-statistics in parentheses, *** *p* < 0.01, ** *p* < 0.05, * *p* < 0.1.

**Table 9:** Robustness test regression results for the baseline regression.

	Model 20	Model 21	Model 22	Model 23	Model 24	Model 25	Model 26	Model 27
*FDI*	0.011 **	0.013 **	0.016 ***	0.017 **	0.017 **	0.015 **	0.015 **	0.013 **
	(2.087)	(2.233)	(2.793)	(2.767)	(2.623)	(2.141)	(2.142)	(2.294)
*rTrl*		0.663 *	0.849 *	0.849 *	1.029 **	0.823 *	0.802 *	0.968 **
		(1.844)	(1.887)	(1.882)	(2.112)	(1.737)	(1.704)	(2.324)
*Pgdp*			0.016	0.014	0.015	0.004	0.004	0.001
			(0.768)	(0.661)	(0.695)	(0.222)	(0.182)	(0.026)
*P*				0.012	0.028	0.024	0.022	0.031
				(0.114)	(0.280)	(0.230)	(0.209)	(0.286)
*Iiffa*					−0.002	−0.002	−0.002	−0.003
					(−0.467)	(−0.409)	(−0.441)	(−0.532)
*Fw*						−0.003	−0.004	−0.006
						(−0.206)	(−0.256)	(−0.410)
*Nofs*							0.001	0.001
							(1.152)	(1.316)
*Fc*								0.005
								(0.900)
Constant	1.336 ***	1.296 ***	1.264 ***	1.210 **	1.122 **	1.174 **	1.188 **	0.998 *
	(32.432)	(24.058)	(20.771)	(2.462)	(2.337)	(2.365)	(2.351)	(1.739)
Regional effect	Yes	Yes	Yes	Yes	Yes	Yes	Yes	Yes
Time effect	Yes	Yes	Yes	Yes	Yes	Yes	Yes	Yes
N	279	279	279	279	268	256	256	256
R^2^	0.705	0.711	0.715	0.715	0.734	0.723	0.724	0.730

Note: Robust t-statistics in parentheses, *** *p* < 0.01, ** *p* < 0.05, * *p* < 0.1.

**Table 10:** Robustness test regression results for moderating effects.

	Model 28	Model 29	Model 30	Model 31	Model 32	Model 33	Model 34	Model 35
*FDI*	0.011 **	0.099 **	0.112 ***	0.111 **	0.113 **	0.102 **	0.101 **	0.088 **
	(2.087)	(2.609)	(2.943)	(2.692)	(2.750)	(2.424)	(2.454)	(2.087)
*rTrl*		0.789 **	1.016 **	1.015 **	1.209 **	0.995 *	0.974 *	1.091 **
		(2.144)	(2.180)	(2.168)	(2.392)	(2.026)	(1.998)	(2.454)
rFdi*Trl		−1.223 **	−1.352 ***	−1.347 **	−1.352 **	−1.225 **	−1.212 **	−1.058 *
		(−2.554)	(−2.820)	(−2.510)	(−2.576)	(−2.319)	(−2.349)	(−1.943)
*Pgdp*			0.018	0.017	0.019	0.008	0.007	0.004
			(0.903)	(0.820)	(0.864)	(0.405)	(0.366)	(0.206)
*P*				0.004	0.020	0.017	0.015	0.023
				(0.034)	(0.200)	(0.157)	(0.139)	(0.210)
*Iiffa*					−0.001	−0.001	−0.001	−0.002
					(−0.281)	(−0.230)	(−0.263)	(−0.364)
*Fw*						−0.004	−0.004	−0.006
						(−0.264)	(−0.306)	(−0.427)
*Nofs*							0.000	0.000
							(1.131)	(1.288)
*Fc*								0.0043
								(0.761)
*Constant*	1.336 ***	1.282 ***	1.251 ***	1.235 **	1.144 **	1.199 **	1.212 **	1.049 *
	(32.432)	(23.690)	(20.144)	(2.487)	(2.364)	(2.385)	(2.370)	(1.783)
*Regional effect*	Yes	Yes	Yes	Yes	Yes	Yes	Yes	Yes
*Time effect*	Yes	Yes	Yes	Yes	Yes	Yes	Yes	Yes
*N*	279	279	279	279	268	256	256	256
*R^2^*	0.705	0.716	0.721	0.721	0.740	0.730	0.731	0.734

Note: Robust t-statistics in parentheses, *** *p* < 0.01, ** *p* < 0.05, * *p* < 0.1.

## Data Availability

Not applicable.
